# Case Report: A Rare Case of Fourth Ventricle to Spinal Subarachnoid Space Shunt Migration: Surgical Pearl and Literature Review

**DOI:** 10.3389/fsurg.2021.696457

**Published:** 2021-07-08

**Authors:** Nicolas Serratrice, Joe Faddoul, Bilal Tarabay, Sarkis Taifour, Georges Naïm Abi Lahoud

**Affiliations:** ^1^ICVNS - CMC Bizet, Paris, France; ^2^CIMOP - CMC Bizet, Paris, France

**Keywords:** cranio-cervical junction, syringomyelia, trapped fourth ventricle, trapped fourth ventricle with syrinx, fourth ventricle to spinal subarachnoid space shunt, catheter migration, Chiari syndrome

## Abstract

**Background:** In the event of syringomyelia communicating with the fourth ventricle, a fourth ventricle to cervical subarachnoid space shunting could be proposed.

**Case Report:** In this review article, we describe the case of a 40-year-old woman who had a previously implanted fourth ventricle to spinal subarachnoid space shunt for the treatment of syringomyelia in the context of Chiari syndrome. The catheter migrated intradurally to the lumbosacral space, but in the absence of neurological repercussions, we decided to leave it in place.

**Conclusions:** To the best of our knowledge, this is the first case described in the literature review of a catheter migration in the subarachnoid space from occipitocervical to lumbosacral level.

## Introduction

Syringomyelia is frequently associated with Chiari syndrome (1, 2). When syrinx cavities communicate with the fourth ventricle, a fourth ventricle to spinal subarachnoid space shunting could be proposed with good clinical results (3, 4). Neurological complications reported with this surgical technique are mainly related to the misplacement of the shunt (5, 6), but the migration of the catheter itself has never been described. In this review article, we report for the first time a case of migration of a subarachnoid catheter from the craniocervical level into the lumbosacral intradural space. We also propose a review of the literature concerning the complications and, in particular, the migration of the different types of shunts used at present.

## Case Report

**Figure 1:**
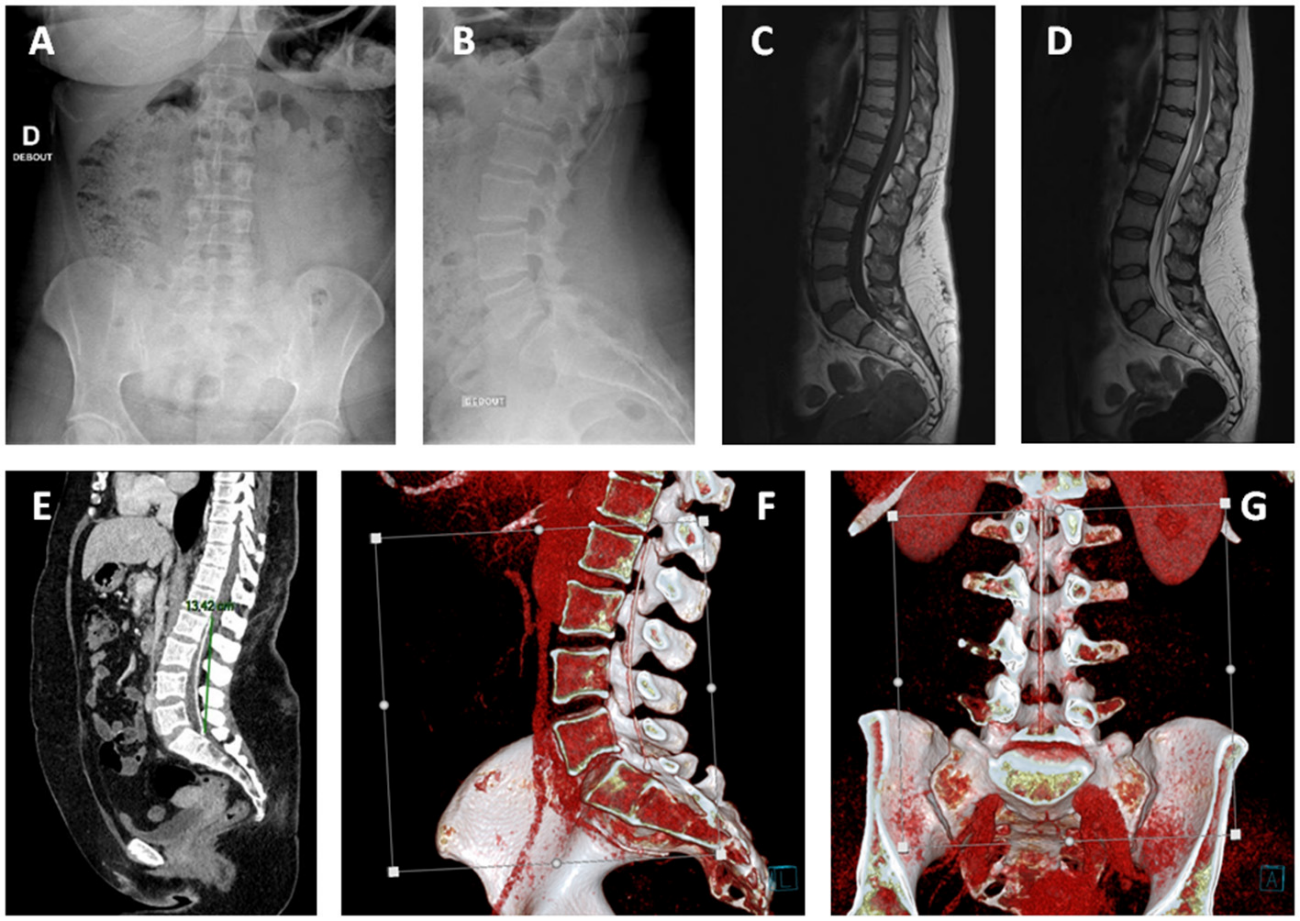
Lumbar x-rays: anteroposterior **(A)** and lateral views **(B)**, MRI sagittal view, respectively, T1 **(C)**, and T2 **(D)** sequences. The presence of the catheter was hard to identify even for experienced radiologists, as its intensity on T1-weighted and T2-weighted images was similar to that of the cauda equina nerve roots. **(E)** Lumbar CT scanner sagittal view showing the initially placed fourth ventricle to cervical subarachnoid space shunt located at L2-S1 level measuring around 15 cm in length and 2.5 mm in diameter. **(F,G)** Three-dimensional (3D) reconstructions. There is no local compression of neurological elements.

**Figure 2:**
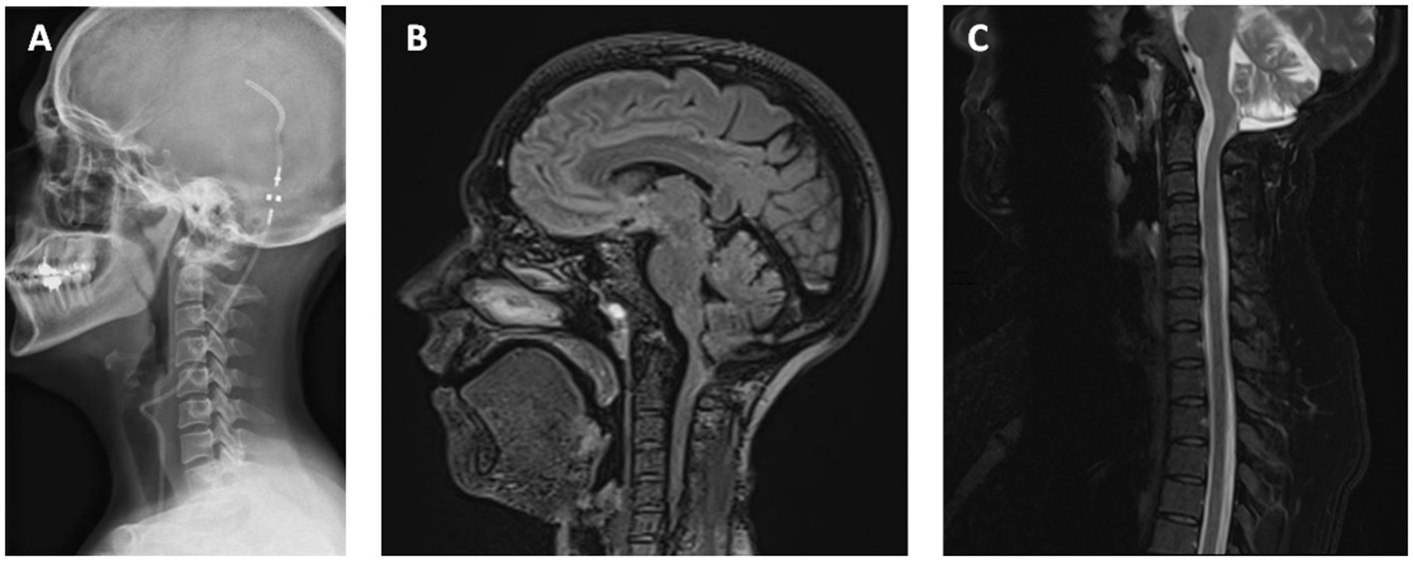
**(A)** Lateral x-ray showing the ventriculoperitoneal shunt. **(B)** No hydrocephaly and dilatation of fourth ventricles on MRI sagittal view T1 sequence. The syrinx has completely regressed **(C)** on MRI sagittal view T2 sequence.

This is the case of a 40-year-old woman who was referred to our institution for the assessment of a complex pathology. She was operated three times for Chiari syndrome between 2010 and 2012. During the second intervention in 2011, a ventriculoperitoneal shunt was placed for neurological deterioration secondary to hydrocephalus. During the last intervention, a fourth ventricle to spinal subarachnoid space shunt with a 15 cm catheter was placed for the progression of syringomyelia associated with a fourth ventricle entrapment. The patient presented significant sequelae including right-side hemiparesis, permanent dizziness, and diffuse chronic pain. In the course of the assessment of recent abdominal pain, the patient benefited from an abdominal CT (Figure 1). The CT showed no intra-abdominal complications. However, the presence of an intradural catheter at the L2-S1 level measuring around 15 cm in length and 2.5 mm in diameter was noted. When looking back to a spine MRI taken in 2018 carried out to investigate mechanical low back pain, the presence of the catheter was hard to identify even for experienced radiologists, as its intensity on T1-weighted and T2-weighted images were similar to that of the cauda equina nerve roots. We concluded that this catheter must have migrated intradurally since it was put in place in 2012. To date, this is the first time such migration of the fourth ventricle to spinal subarachnoid space shunt is described in the literature review. Its presence is not responsible for pain or neurological symptoms as it is well-confirmed clinically and on the electromyogram. The ventriculoperitoneal shunt seemed to be functional, and no fourth ventricle entrapment was found (Figure 2). No residual syringomyelia was detected on the spine MRI. Hence, we found no indication to place a new shunt nor to remove the migrated one.

## Discussion

In this review article, we describe the case of a 40-year-old woman with a history of syringomyelia in a context of Chiari syndrome (1), who had a previously implanted fourth ventricle to cervical subarachnoid space shunt that migrated to the subdural lumbosacral level. Because of the absence of syringomyelia and ventricular dilation, as well as the absence of neurological repercussions, we decided to leave the catheter in place.

Several types of shunts can be used in the treatment of syringomyelia, namely, fourth ventricle to cervical subdural, syringo-subarachnoid, syringo-peritoneal, syringo-pleural, lumboperitoneal, and ventricular shunts. Each of these techniques has its well-known advantages and complications. Among the complications, catheter migration remains extremely rare. However, to be safe and effective, it is recommended to perform such techniques selectively by an experienced neurosurgeon.

In 1995, Lee et al. reported five complications on 12 patients treated by fourth-ventricular shunting for symptomatic posterior fossa cysts of the Dandy-Walker malformation and trapped the fourth ventricle: Three patients developed new cranial nerve dysfunction caused by direct injury to the floor of the fourth ventricle, one patient suffered an intracystic hemorrhage and acute shunt malfunction, and one patient had the catheter tip in the brainstem on postoperative studies without new neurological deficit (5). These complications were mainly related to the misplacement of the catheter, and even if the techniques have evolved, options include, nowadays, the following: open fenestration through a suboccipital craniotomy, fourth-ventricular shunting, and minimally invasive procedures including endoscopic stenting and fenestration. These techniques are associated with complications in 42% of cases (6–10).

Trapped fourth ventricle with or without syringomyelia is a rare condition, and there is still no consensus on its management strategy. However, in this context of migration of the fourth ventricle to subarachnoid space shunt, the ventriculoperitoneal shunt previously implanted might have in itself induced the long-term resolution of the syringomyelia. This would, therefore, suggest the limited impact of the fourth ventricle to subarachnoid space shunting. Such a view is also shared by a growing portion of the adult and pediatric neurosurgical community (11).

Syringomyelia associated with spinal arachnoiditis or arachnoid adhesions can be treated by adhesiolysis using a conventional microscopic approach or an endoscopic technique in order to place a spinal cysto-subarachnoid shunt (12).

A thorough neuroradiological workup is the best way to ensure the least number of perioperative and postoperative complications and maximal chances of satisfactory long-term clinical improvement in Chiari syndrome (13). When there is persistence, recurrence, or progression of the syrinx after a foramen magnum decompression (30% of the patients), syringomyelia can be treated with a syringo-subarachnoid shunt (14). However, rare neurological complications have been reported (15).

Complications of migration of lumboperitoneal shunt are frequent (16, 17). General complications are similar to ventriculoperitoneal shunt, i.e., abdominal pseudocyst, distal catheter migration, inguinal hernia, catheter disconnection, infection, and intestinal obstruction (18).

## Conclusions

To our knowledge, this is the first described case of catheter migration from the fourth ventricle to the lumbar subarachnoid space in the context of Chiari syndrome associated with syringomyelia. Because of the absence of radiological and neurological repercussions, we decided to leave the catheter in place.

## Data Availability Statement

The original contributions presented in the study are included in the article/supplementary material, further inquiries can be directed to the corresponding author/s.

## Ethics Statement

Ethical review and approval was not required for the study on human participants in accordance with the local legislation and institutional requirements. The patients/participants provided their written informed consent to participate in this study.

## Author Contributions

NS: investigation, writing—original draft, and visualization. JF: writing—review and editing and visualization. BT and ST: writing—review and editing. GNAL: supervision and writing—review and editing. All authors contributed to the article and approved the submitted version.

## Conflict of Interest

The authors declare that the research was conducted in the absence of any commercial or financial relationships that could be construed as a potential conflict of interest.
